# Evidence for mood-dependent attentional processing in asthma: attentional bias towards health-threat in depressive mood and attentional avoidance in neutral mood

**DOI:** 10.1007/s10865-018-9919-6

**Published:** 2018-04-06

**Authors:** Iana Alexeeva, Maryanne Martin

**Affiliations:** Medical Sciences Division, Department of Experimental Psychology, University of Oxford, Anna Watts Building, Radcliffe Observatory Quarter, Woodstock Road, Oxford, OX2 6GG UK

**Keywords:** Attention, Avoidance, Cognitive processes, Depression, Asthma, Health

## Abstract

Attentional biases have been observed in populations with psychological disorders, but have been under-investigated in populations with physical illnesses. This study investigated potential attentional biases in asthma as a function of mood. Asthma (N = 45), and healthy (N = 39) participants were randomly allocated to a depressed or a neutral mood state induction. They completed a visual probe task that measured participants’ reaction times to health-threat and neutral pictures and words. Compared to the healthy controls, the asthma group showed attentional bias towards health-threat pictures in depressed mood, and avoidance of health-threat pictures in neutral mood. Attentional biases were found in a group with a physical illness as a function of induced mood. It is suggested that attentional processes in people with physical illness may be important in relation to symptom perception and illness management.

## Introduction

Current asthma treatments aim to control and manage the symptoms in order to prevent lung function deterioration and asthma attacks (GINA 2015). In spite of effective medication that allows individuals to function close to full capacity, there are high rates of uncontrolled or partly controlled asthma (Peters et al., 2006; Thomas et al., 2009), resulting in symptom exacerbation and elevated morbidity (Humbert et al., 2007).

Generally, asthma is a condition managed by people themselves rather than by healthcare professionals (McWilliam, 2009; Newman et al., 2008). It is particularly important, in order to ensure well-controlled and well-managed asthma, that individuals can implement the routine use of effective and relatively non-invasive medication, and this is a process influenced by the individual’s thoughts and actions (Kaptein et al., 2010). Thus, one of the current research aims in asthma is to understand how cognitive, emotional, or behavioural processes influence the decisions that people make concerning management and treatment of their illness.

Symptom monitoring may be an adaptive response in managing a chronic condition. Self-monitoring was found to be a protective and adaptive strategy in diabetes (O’Kane et al., 2008) and weight control (Baker & Kirschenbaum, 1993). Asthma, for example, requires adherence to a daily medication regime an early and quick detection of symptom escalation to prevent an asthma attack. One of the ways to achieve effective symptom control and management is by monitoring lung function and somatic symptoms, using objective and subjective measures. It has been suggested that in respiratory disease some degree of illness-specific concern and monitoring may be helpful for early symptom detection and effective management (De Peuter et al., 2004). It is the extreme ends of the monitoring process, either over- or under-perception, that may be maladaptive. For example, in case of under-perception, failing to detect a rise in symptoms may lead to a potentially fatal asthma attack. Conversely, in case of over-perception, external and internal cues may lead to increased sensitivity to symptoms (e.g. breathlessness) and amplified perception of symptoms in the absence of any physiological changes, within the asthma population (De Peuter et al., 2007).

It is clear how under-perception of symptoms may be a disadvantage in asthma, if it can put an individual at risk of failing to prevent symptom exacerbations or an asthma attack, or adhere to medication (Davis et al., 2009; Magadle et al., 2002). Over-perception is a little harder to envisage as a maladaptive strategy in asthma. It is more difficult to draw the line between adaptive extra-monitoring needed in managing a serious chronic physical condition and the kind of over-perception associated with negative outcomes. De Peuter et al. (2007) suggested that symptom over-perception by those with a chronic illness may be one of the risk factors for negative health outcomes and symptom perpetuation; in asthma specifically, over-perception of symptoms may contribute to impaired asthma control. In a similar vein, men with HIV and women with cervical dysplasia demonstrated greater catastrophic and negative beliefs and consequent avoidant coping if they showed high monitoring tendencies (Miller et al., 1996).

Janssens et al. (2009) proposed that the answer to poorly controlled asthma may lie in a better understanding of the cognitive-emotional processes underpinning symptom perception. They argued that perceptual accuracy is underpinned by mechanisms that integrate top-down (cognitive representations of illness, an illness schema) and bottom-up (current sources of sensory, affective, and contextual information) processes. Janssens and colleagues imply that attentional processes may be vital to symptom perception, evaluation, and consequent decisions regarding symptom management. Attention may be utilised in closer monitoring of external and internal information, leading to over-perception, or attention may be diverted away, resulting in avoidance and under-perception. Thus, biased attentional processing may explain inaccurate symptom perception and underpin the discrepancies between subjective experience of asthma symptoms and the objective measures of lung function.

Attention is the initial step in the monitoring process, and is proposed to consist of three stages: attentional orienting, attentional capture or engagement, and disengagement or shift towards a different information source (Posner & Petersen, 1990). Attentional bias implies preferential processing of certain information, for example a preference for negative information in the context of emotional disorders (Mathews & MacLeod, 2005). Logically, the experimental paradigms and theoretical models of biased attentional processing have been most developed in anxiety research.

Different facets of attentional bias have been identified: hypervigilance—alerted state and amplified attentional focus on threat; difficulty in disengagement—impairment in shifting attention away from threat; avoidance—directing attention away from threat without meaningful engagement or deeper processing (Cisler & Koster, 2010; Koster et al., 2006; Mathews & MacLeod, 2005). Experimental paradigms that assess the course of attention include the modified Stroop, the visual probe, and spatial cueing tasks (Cisler & Koster, 2010). The advantage of these tasks is the objective assessment of selective attentional processing by measuring the degree of interference between task irrelevant threat-related stimuli and task performance, thus preventing the influence of response bias (MacLeod et al., 1986).

The modified emotional Stroop task involves naming the colour of words that vary in emotional meaning. It is hypothesised that if the words are threatening (pain, illness, death) then it slows down the colour naming. If participants are slower to colour-name threat words as opposed to neutral or positive words, then the biased attention towards threat is inferred (Mathews & MacLeod, 2005). In health research, studies using the Stroop task demonstrated that individuals who experienced recent minor illness were slower to colour-name words related to illness and health than general threat or neutral stimuli (Karademas et al., 2008). There is also evidence for threat interference in chronic pain, however it is inconsistent and difficult to interpret (Crombez et al., 2013; Pincus & Morley, 2001; Roelofs et al., 2002; Schoth et al., 2012). In asthma there is some evidence of Stroop interference for asthma-related words rather than for negative emotional words (Jessop et al., 2004; Martinez-Moragon et al., 2003). However, the study by Martinez-Moragon et al. (2003) did not include a control or a comparison group, and it cannot be claimed that there is biased cognitive processing specifically characteristic of asthma. Jessop et al. (2004) compared an asthma group to a healthy control group and to a group consisting of healthy people who were primed about asthma, having read a few excerpts from interviews with people with asthma, prior to completing the task. The stimuli in the study included asthma-related, negative emotional, and neutral words. The asthma group showed greater interference for asthma-related words, compared to both groups. Moreover, for the asthma group the interference from asthma words was greater than the interference from negative emotional words. Hence, the evidence from studies using the Stroop task points towards increased salience of asthma-related information for people with asthma, however that does not necessarily suggest a presence of a maladaptive attentional bias.

One of the problems identified with the Stroop task is that it is difficult to separate the interference with the colour-naming because of attentional bias towards threat from the cognitive avoidance of unpleasant stimuli (de Ruiter & Brosschot, 1994). Furthermore, there is a problem of identifying the specifics of attentional bias at different stages, because the difference in colour-naming latencies could result from either participants’ orienting faster to threat stimuli, or having difficulty, once drawn to threat, in disengaging from it (Fox et al., 2001). Another criticism of the Stroop task is that it measures emotional processing rather than visuo-spatial attention process, hence the problem of the separation of the bias at each attentional stage (Hou et al., 2008; Mathews & MacLeod, 2005).

Employing alternative methods, like a visual probe task, may tap into different facets of attention. MacLeod et al. (1986) developed a visual probe task that measures participants’ reaction times to various types of stimuli presented visually on a computer screen. The paradigm is thought to measure components of visual-spatial attention. Reaction times are faster to attended locations, therefore the task can measure the allocation of attention towards threatening or neutral stimuli and the magnitude of attentional bias towards a particular type of information.

In a visual probe task a pair of stimuli (words or pictures) is presented in two different locations, typically one word on the top half of a computer screen and the other on the bottom. One stimulus in a pair represents a threat (e.g. physical, emotional), the other stimulus in a pair is neutral, for example, it can be a word pair “death—table”. Then a visual probe (typically a dot) appears in the same place as one of the stimuli presented previously. The trials may be congruent, where the dot replaces a threat stimulus, or incongruent, where the dot replaces a neutral stimulus. This is one of the advantages of the task, because it allows calculation of congruence and incongruence indices corresponding to, respectively, attentional engagement and difficulty in disengaging attention from threat (Koster et al., 2004; Koster et al., 2006).

Moreover, in the visual probe task the exposure times can be varied in order to tap into different stages of the attentional process: an automatic preconscious stage can be assessed with stimuli exposure times up to 500 ms, while higher order, strategic voluntary allocation of attention can be measured with longer stimulus duration of 500 ms and up to 1250 ms (Mogg & Bradley, 1999; Mogg et al., 2004).

Additionally, the task difficulty can be varied allowing observation of attentional processes in more detail under different cognitive loads. A more difficult version of the task involves identifying the type of probe, for example whether an arrow is pointing left or right, rather than indicating a simple position of the probe such as top or bottom of the screen. The identification version is thought to be more cognitively demanding and more difficult to learn because of the lack of direct stimulus–response mapping (Mogg & Bradley, 1999; Schoth et al., 2012). However, it has an advantage in encouraging a more even monitoring of visual space, because the probe appears an equal number of times in all locations, thus preventing bias towards a particular region of space (Mogg & Bradley, 1999). Mogg and Bradley, having compared the two types of tasks, showed that probe identification task may produce more errors and slow down the response times. However, the task does not interfere with the attentional pattern of responses (Mogg & Bradley, 1999), and it is deemed more important to ensure an even monitoring of the screen in order to distinguish the attentional bias towards particular stimuli from the adapted biased response of monitoring a particular region of space. Furthermore, the attentional bias may become evident when the attentional resources are strained during a more cognitively demanding task. It may be difficult to detect attentional bias if the task is too easy and the patients have already developed strategies in managing their attentional resources in a chronic condition, for example chronic pain (Pincus & Morley, 2001).

The visual probe task has been employed extensively in chronic pain research and provided a coherent account of attentional processes in pain. The empirical observations in chronic pain appear to be consistent with the difficulty in disengagement from pain stimuli at later attentional stages (Dehghani et al., 2003; Roelofs et al., 2005). Schoth et al. (2012) provided evidence that attentional bias occurs in the later stages of attention, around 1250 ms mark. The findings were also confirmed by a meta-analysis by Crombez et al. (2013) who found a significant effect size for chronic pain patients when the stimuli were presented for longer than 1000 ms. Even though the evidence for earlier attentional bias and hypervigilance in chronic pain is less consistent, coming from the laboratory-based studies using Stroop, visual probe, or spatial cueing paradigms (Schoth et al., 2012; Crombez et al., 2013) the eye-tracking evidence suggests the presence of greater early vigilance for pain-related stimuli in chronic pain patients and people with elevated fear of pain (Liossi et al., 2014; Yang et al., 2013; Yang et al., 2012). Todd et al. (2015) also detected a pattern of early initial vigilance, which was followed by a pattern of attentional avoidance.

Todd and colleagues’ findings may contradict the results of the meta-analyses by Schoth et al. (2012) and Crombez et al. (2013) which pointed towards difficulty in disengaging from pain stimuli rather than a hyper-vigilance avoidance pattern detected by Todd et al. It is important to note two potential explanations for the mixed findings. One involves the differences in populations studied and the second involves issues with the methodology. The meta-analyses by Schoth et al. and Crombez, Van Ryckeghem, et al. covered the findings from chronic pain patients, while Todd and colleagues investigated a mix of acute pain, chronic pain, or currently pain free individuals expecting a medical procedure. As for the methodology, the visual probe is an incredibly flexible tool where one can vary stimuli type, duration, intensity, and presentation methods. It is a great advantage when one is aiming to explore the intricacies of attentional processing, but may make difficult comparison between different groups, studies, populations, or settings resulting in inconsistent findings (Schmukle, 2005).

There have been very few studies in asthma that used a visual probe task. The findings, not dissimilar to chronic pain, are mixed, and the inconsistencies may stem from similar issues: differences in observed populations and methodological differences. The studies so far have involved adult and children populations, word stimuli specifically related to asthma, and varying stimuli presentation times. Therefore, it is too early to make firm conclusions with regard to the presence, magnitude, or specific nature of the attentional bias in asthma, but utilising the visual probe task may aid in the exploration of the facets of attentional processing in asthma.

De Peuter et al. (2007) used a visual probe task with a 500 ms stimulus duration. The stimuli included asthma-related words and general negative words. The study did not detect a significant difference between the asthma group and healthy controls in their response to asthma-related words. Although, the researchers observed that within the asthma group attentional bias towards asthma words was related to greater negative affect. Their negative word list was quite varied including health-threat (e.g. cancer), physical threat (e.g. crime), and social threat (e.g. insult). It is possible that there was enough of an overlap with regard to illness-related information between the asthma-specific and general negative stimuli that the task did not distinguish between attending to asthma-related or to general negative stimuli.

The other two studies on attentional bias in asthma were conducted with children (ages 8–13), and present conflicting evidence. Lowther et al. (2015) found attentional bias towards asthma words in children with asthma as compared to children without asthma using a visual probe task that included asthma-related, anxiety, and negative emotional words. These words were presented for 1250 ms, deemed more suitable for children because their processing times are likely to be slower than adults. Lowther and colleagues detected an attentional bias for asthma words in the asthma group, but not for anxiety or negative emotional words. The authors compared congruent and incongruent trials within the asthma group and found no difference, therefore making no claim whether the bias in the asthma group for asthma words occurred earlier or later in the attentional processing.

In contrast, Dudeney et al. (2017) did not observe an attentional bias towards asthma words in children with asthma despite replicating the method used by Lowther et al. (2015). The study compared an asthma group, with a group with comorbid asthma and anxiety, a group with anxiety alone, and healthy controls. Asthma-related and general negative words were presented for 1250 ms. The general negative word list included physical and social threat (e.g. bully, murder, loser). Dudeney and colleagues did not detect group differences, word type bias differences, or significant interactions. Their results are even more surprising with regard to their anxiety group. Attentional bias findings in anxiety are reasinably robust (Bar-Haim et al., 2007), and thus one would expect at least an attentional bias towards general threat in children with anxiety. More reassuringly, the study did find a significant association between attentional bias and level of anxiety in the asthma group. The lack of significant group effects may be due to their study being somewhat underpowered (the sample sizes per each group were quite small, 15–29 per group). Alternatively, Dudeney and colleagues suggest that there were varying levels of asthma severity in the two studies. Lowther et al’s investigation focused on moderate to severe asthma whereas Dudeney and colleagues’ study included children with varying levels of asthma.

Another potential explanation for mixed findings of De Peuter et al. (2007) and Lowther et al. (2015) is that an illness schema in children may be different from a perhaps more established schema in adults. In a similar vein, Pincus and Morley (2001) suggest that people with long-term chronic pain have already developed methods of coping with their pain which would be reflected in their attentional processes with regard to pain-related information. Asthma is frequently diagnosed in childhood. Therefore, we may be observing the early processes of establishing an illness schema and its interaction with cognition in children, which is less likely to be reliable and stable, and more likely to be changing within the context of various developmental processes.

Importantly, and in line with theories with regard to the impact of symptom perception processes in asthma, Dudeney et al. (2017) did find a significant relationship between poorer asthma control (parent reported asthma control problems) and increased attentional bias in children with asthma. The researchers consequently discussed whether the relationship between poorly controlled asthma and attentional bias is adaptive or maladaptive. On the one hand, the control may be poor because the asthma is very severe, and attentional bias towards asthma-related information may be adaptive if one’s asthma is severe and demands greater effort in controlling it. Increased attention may also aid symptom monitoring. On the other hand, increased asthma severity may be due to poor control and inaccurate symptom perception underpinned by attentional bias, which may undermine control and contribute to increased symptom severity.

Thus, empirical observations so far point towards potential attentional bias in asthma, primarily focused on asthma-related stimuli as opposed to more general physical threat, or negative emotional stimuli. Current evidence, however, does not allow a confident prediction as to whether the bias will be observed at earlier or later attentional stages due to hypervigilance or difficulty in disengagement. The duration of stimuli presentation in the current study was set at 500 ms to replicate the methodology employed by De Peuter et al. (2007), the only other study using visual probe task with adults with asthma allowing for a better comparison.

Even though the past findings with regard to attentional bias in asthma are mixed, there is one observation that appears to be quite consistent, and that is the correlation between greater attentional bias and negative affect. The study by De Peuter et al. (2007) points towards the reciprocal relationship between attention and mood, rather than pure attentional bias in asthma. Negative affect and depressed mood in asthma are associated with attentional bias towards respiratory-related words, increased sensitivity to symptoms (e.g. breathlessness) and amplified perception of symptoms in the absence of any physiological changes in adults (De Peuter et al., 2007; Jessop et al., 2004; Martinez-Moragon et al., 2003; Put et al., 2004) and in children (Lowther et al., 2015). To add to that picture, asthma has a high comorbidity with depression (Trojan et al., 2014), and depression in asthma is associated with poorer symptom control (Lavoie et al., 2006). Thus, it is possible that depressed mood may exert an influence on symptom perception and control via the mechanism of biased attention towards symptom-related information and health-threat. Even though the asthma studies discussed above have provided initial evidence for the relationship between negative mood and attentional bias in asthma, that relationship was not the focus of those studies. There have not yet been in depth investigations into the association between negative mood and attentional bias, in spite of the reciprocal relationship between emotion and cognitive processing suggested by theories of asthma symptom perception (Janssens et al., 2009, 2012).

Janssens et al. (2012) proposed that maladaptive over-perception is more likely to have an effect on symptom exacerbation in interaction with negative affect, anxiety, panic, and fear, as well as avoidance. In a similar vein, theoretical accounts of medically unexplained symptoms propose that negative mood and emotional distress may lead to increased attention towards symptoms, illness worry and catastrophic interpretation of the symptoms, thereby maintaining negative illness cognitions and perpetuating the symptoms (Brown, 2004; Kolk et al., 2003). Evidence obtained from people with chronic pain indicates that negative emotions can bring about more fearful thoughts about pain and self-reported attentional focus on pain was higher in the moments when people experienced more intense pain, elevated fear of pain, and reduced positive emotions (Crombez et al., 2013). Among healthy people, inducing a ruminative state resulted in greater attentional vigilance towards affective pain words (Brookes et al., 2017). Rumination involves automatic negative repetitive thinking about something, thought to trigger a state of worry and catastrophizing, and strongly associated with depressed mood (Nolen-Hoeksema, 2000; Nolen-Hoeksema et al., 2008).

Therefore, negative mood may play a role in directing attention towards internal sensations or external cues. It may be responsible for activating an illness schema and increasing attentional bias, thus leading to symptom overperception and undermining asthma control. Consequently, the current study employed a mood induction technique in order to investigate a direct influence that depressed mood may have on attentional bias in a sample of adults with asthma with no current comorbid mental health disorders (such as depression or anxiety). Factoring mood in would also allow a more concrete prediction regarding where in the attentional process a bias may occur. In depression, attentional bias tends to manifest at a later voluntary elaborative stage, as in difficulty in disengaging from negative stimuli (Gotlib & Joormann, 2010). Therefore, we can make a prediction that should the attentional bias in asthma in depressed mood be observed, it is more likely to be driven by difficulty in disengagement rather than hyper-vigilance.

Another important matter concerns threat severity and relevance to current illness concerns. People with social phobia showed attentional bias towards specific phobia-related words, in comparison to those with generalized anxiety, who showed broad-spectrum attentional bias towards all non-specific threat (Becker et al., 2001). Stimuli that are highly relevant to individuals’ concerns in specific disorders may activate an illness schema congruent with the threat stimuli leading to attentional bias (Martin et al., 1991; Williams et al., 1997). Henderson et al. (2007) showed that even healthy people, if primed with a specific illness schema (e.g. cardiovascular disease), demonstrated attentional bias towards the health-threat stimuli directly relevant to that specific illness schema, cardiovascular disease as opposed to the common cold. Chronic pain patients are more likely to show attentional bias towards more specifically sensory pain words, as opposed to, for example, affective pain stimuli (Crombez et al., 2013). As reviewed above, the evidence from studies of asthma suggests that attention is directed towards asthma-related stimuli. However, as discussed by Dudeney et al. (2017), the question of how adaptive or maladaptive an attentional bias may be in asthma remains open. A certain degree of attention towards asthma-related information is needed for accurate symptom perception (De Peuter et al., 2004; Rietveld, 2003).

What may turn an adaptive monitoring process into a maladaptive attentional bias undermining symptom control and management? It is possible that a process of overgeneralisation may play a role. As long as attention is focussed on asthma-related information, symptom perception may remain relatively accurate. However, if attentional bias is extended to more general illness or health-related information, covering a greater variety of internal and external cues, then over-perception may become an issue and undermine the process of adaptive monitoring.

Put et al. (2004) showed that people with asthma and high negative affectivity reported more intense asthma symptoms following a suggestion of increased bronchoconstriction, and were similarly susceptible to the suggestion of bronchodilation, reporting lower airway obstruction symptoms post-suggestion. Put and colleagues argued that those high in negative affect may be using more than strictly asthma-related cues to interpret their internal state. Livermore et al. (2007) demonstrated an attentional bias among people with chronic obstructive pulmonary disease towards physical threat words, rather than illness-specific, respiratory stimuli. Though, Livermore and colleagues also showed similar bias in their healthy control group, which might have been due to the highly threatening nature of the stimuli they used, which they borrowed from the anxiety literature.

Consequently, the current study utilised more general health-threat words and pictures, instead of specifically asthma-related stimuli, in order to further investigate the nature of attentional bias in asthma. We hypothesised that attentional bias in asthma extends beyond asthma-specific stimuli towards more general potential health-threat. However, the stimuli were chosen to represent strictly health-related threat rather than negative affect, or physical or social threat.

Additionally, exercise has long been recognised as a potential trigger for inducing asthma symptoms or an asthma attack (Parsons & Mastronarde, 2009). People with asthma may perceive exercise as a health-threat, as a potential source of danger to their established health/illness balance and their management of asthma symptoms. Physical activity, particularly exercise, is associated with fear cognitions in people with asthma (Emtner et al. 1996). Physical activity has direct relevance and may be highly salient to people with asthma, and therefore an attentional bias towards activity as health-threat would be expected to occur. Attentional bias towards activity-related stimuli may similarly show the process of overgeneralisation leading to overperception.

Previous visual probe studies in asthma used linguistic stimuli. There has been some concern over the ecological validity of words, which may be too limited in context and meaning (Mogg & Bradley, 1999). Pictures were suggested to be more relevant than words for illness groups (Moss-Morris & Petrie, 2003). There is also a problem with linguistic stimuli in the sense that participants may have varying degree of familiarity with particular words, even if they are matched on frequency of use. This is a problem that does not apply to pictures (Mogg & Bradley, 1999). Therefore, the current study used both linguistic and pictorial stimuli to investigate the attentional bias in asthma. If pictures are more ecologically valid they would be more likely to activate an illness schema. It is therefore expected to observe stronger attentional bias in response to pictures rather than words.

Thereby, the current study focused on a key cognitive process: attention, which was measured via an objective experimental task, the visual probe task. In order to investigate a direct effect of mood on attention, the current experiment employed the Velten mood induction procedure (Velten, 1968). Participants were allocated to undergo either depressed or neutral mood induction before completing the attentional task. Stimuli were presented for 500 ms to allow the measurement of conscious attentional processing and the voluntary allocation of attention towards threatening stimuli. The following hypotheses were tested:

1. The asthma participants following depressed mood induction will show greater attentional bias towards health-threat words and pictures compared to the asthma participants in neutral mood, who in turn will show greater attentional bias compared to the healthy controls. We expect to observe significantly higher scores on attentional bias index in the depressed asthma group.
2. The asthma participants, following depressed mood induction, will demonstrate greater difficulty in disengaging their attention from threat stimuli, indicated by a slower response to a probe replacing neutral stimuli on neutral-threat trials, compared to neutral–neutral trials.
3. The asthma participants following depressed mood induction will demonstrate greater attentional bias towards threat pictures than threat words.

## Method

### Participants

Participants were recruited from Oxfordshire communities and clinics via posters, online and newspapers advertisements. The inclusion criteria for the participants in the asthma group were that they a) reported a primary asthma diagnosis by a GP or referred specialist, b) had no other current acute or chronic medical or mental health diagnosis, and c) were prescribed a reliever inhaler. Participants reported illness duration (months/years), severity, and daily asthma interference with functioning on a scale from 0 (not at all interfering) to 100 (extremely interfering) (Table 1). Healthy controls were excluded if they reported a current acute or chronic medical or mental health diagnosis. All data exclusions, manipulations, and measures in the study are reported below.

**Table 1:** Demographic and questionnaire measures for asthma and healthy control groups

	Asthma	Control	*χ2*	*p*
	(n = 45)	(n = 39)		
	*n* (%)	*n* (%)		
Sex (female)	27 (60%)	18 (46%)	1.6	*ns*
Education (degree)	33 (72%)	28 (61%)	0.5	*ns*

	*M* (SD)	*M* (SD)		
Symptoms duration (years)	20.7 (13.1)			
Time since diagnosis (years)	20.0 (13.2)			
Asthma severity (0–100 scale)	41.2 (25.9)			
Asthma interference (0–100 scale)	28.3 (25.6)			

	*M* (SD)	*M* (SD)	*F*	*p*
Age	32.4 (11.1)	36.9 (12.7)	3.1	*ns*
HADS depression	5.3 (3.9)	4.6 (3.4)	0.7	*ns*
HADS anxiety	6.0 (4.1)	7.0 (3.5)	1.4	*ns*

*HADS* Hospital Anxiety and Depression Scale

### Self–report measures

Anxiety and depression were measured using the Hospital Anxiety and Depression Scale (HADS), a 14 item scale for populations with non-psychiatric medical conditions, so that the scores are not affected by the physical symptoms of the medical condition (Zigmond & Snaith, 1983). The range of scores is from 0 to 21 for each subscale, with a higher score indicating greater severity. The developers suggest the following classification of cases based on the scores: normal range (0–7), mild (8–10), moderate (11–15), and severe (16 or higher). The scale has good reliability in the current sample, Anxiety subscale Cronbach’s α = 0.81 and Depression subscale Cronbach’s α = 0.75.

The Positive and Negative Affect Schedule (PANAS) assessed momentary mood (Watson et al., 1988). Participants read 10 adjectives (e.g. anxious) and indicated to what extent they were feeling that way right now, using a 5-point scale (1-very slightly or not at all to 5-extremely). In the current sample, Cronbach’s α was 0.90 for the Positive Affect (PA) subscale and 0.79 for the Negative Affect (NA) subscale.

### Mood induction

The mood induction, a commonly used, well-validated procedure lasting 8 min (Martin, 1990; Moore & Oaksford, 2002) included explicit instructions, music, and Velten statements (Velten, 1968). The mood induction procedure took place before the computer task. Participants were randomly allocated either to a depressed or a neutral mood state induction. In each condition participants were instructed to read through a list of statements for 8 min while listening to music. They were asked to read slowly to themselves, use their imagination and concentration to focus on each of the ideas represented in the sentences, and to visualize them in turn. In the depressed mood condition participants listened to music (Barber, Adagio for Strings) (Moore & Oaksford, 2002) while reading a list of statements describing feelings of sadness, loneliness, unhappiness (e.g. ‘It often seems that no matter how hard I try, things still go wrong.’). In the neutral mood induction, participants listened to Brahms 2nd movement from the 3rd symphony while reading about historical facts or descriptions of objects (e.g.‘West Samoa gained its independence in 1965.’). Participants’ mood was assessed using PANAS before and after the induction.

### Visual probe task

The visual probe task was adapted from previous attention experiments (Lees et al., 2005). An experimental trial started with a presentation of a central fixation cross (size 14 mm × 14 mm) for 500 ms on a computer screen. It was followed by a word- or picture-pair presented for 500 ms, with one cue above the fixation point and another cue below. For the word pairs, their vertical distance from the fixation point was 50 mm, so the distance between their inner edges was 100 mm. The words varied in size depending on their length, ranging in length 40–95 mm and height 6–10 mm. The picture size and positioning were replicated from the previous experiments in anxiety (Lees et al., 2005). The pictures measured 70 mm in height and 96 mm in length. The distance between their inner edges was 50 mm.

**Fig. 1:**
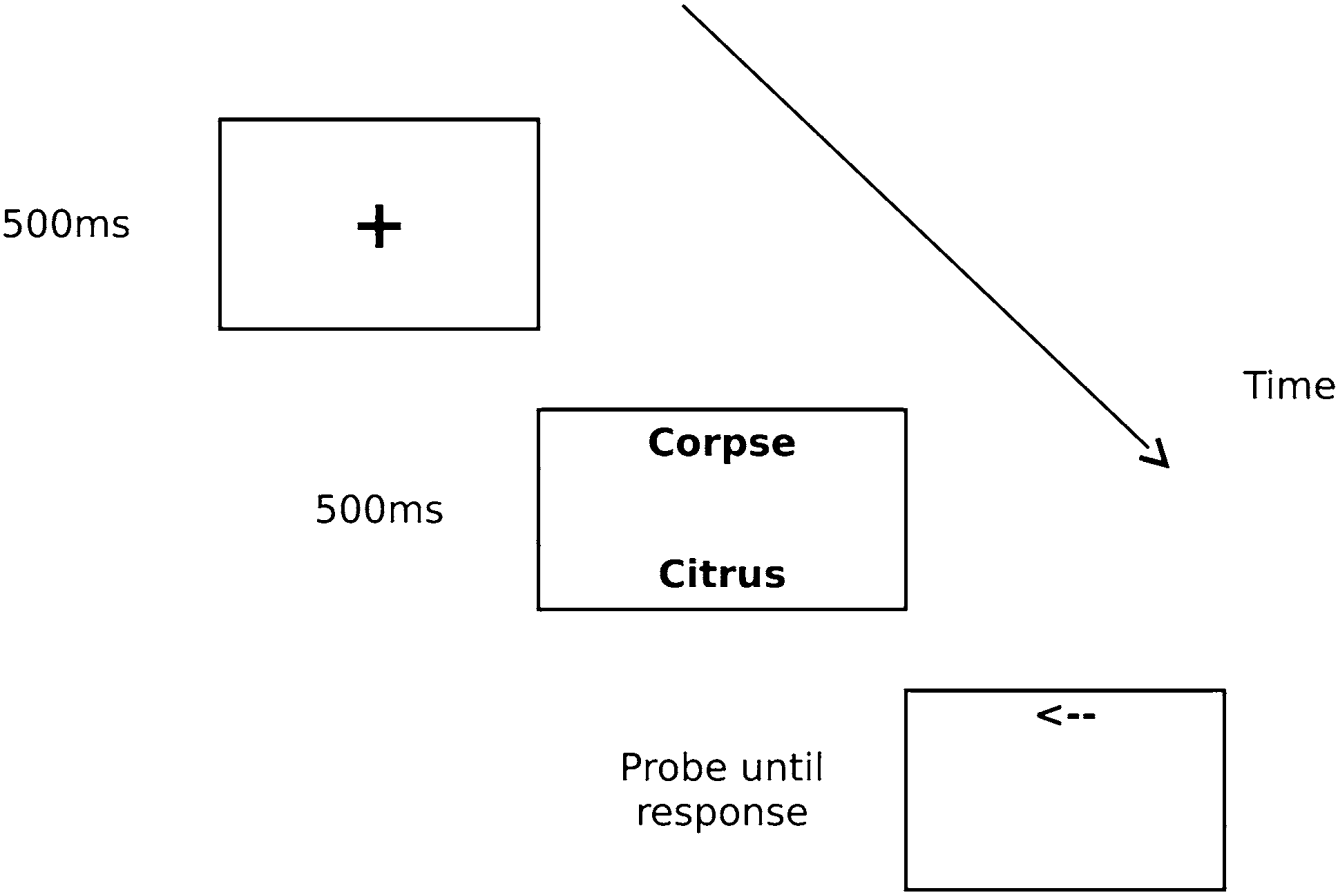
The sequence of stimuli presentation in the Visual Probe task

The stimulus pair was followed by a 50 ms blank mask. Then the arrow (length 15 mm) appeared either above or below the fixation point (vertical distance from the fixation point was 46 mm) and stayed on the screen either until participants responded or for 2500 ms. The inter-trial interval was 1000 ms. Each threat cue could appear in one of two positions (above or below the fixation point) and an arrow replaced the threat cue or the neutral cue. Figure 1 presents a graphic representation of an experimental trial, a probe replacing a threat stimulus.

The following instructions were given to the participants: to look at the fixation cross on the monitor, and when an arrow appeared on the top or bottom half of the screen, press a key on the computer keyboard to indicate whether the arrow pointed to the left (**z** key) or to the right (**m** key). Participants were instructed to concentrate on detecting the arrows and not the words or pictures, which might have preceded them. They were asked to be as fast and as accurate as possible and maintain their gaze at the centre of the screen.

Participants received instructions and completed a practice block (16 trials, 8 word and 8 picture trials), after which they had the opportunity to ask any questions or rerun the practice. Each experimental word or picture pair was presented four times, so that each threat cue was presented in two positions (above and below the fixation point) and the arrow replaced the threat cue (valid trial) or the neutral word (invalid trial). Therefore, the presentation of the 48 pairs (16 of each: Health Threat–Neutral, Activity–Neutral, Neutral–Neutral) produced 192 experimental word trials and 192 pictures trials; in total, 384 experimental trials. Of those trials, half had the arrow pointing left, and half had the arrow pointing right. Trials were presented in a random order, where the word and picture trials were intermingled. The task took on average 14 min to complete.

### Stimuli

The 48 word-pairs, 16 health-threat and neutral (e.g. cancer-feline), 16 activity and neutral (e.g. jogging-chrome), 16 neutral and neutral (e.g. flute-flint), were selected from previous research on attention (Lees et al., 2005). In addition 8 neutral word pairs and 8 neutral picture pairs were created for use in the practice trials. Words were matched on length and frequency of occurrence (Carroll et al., 1971), all *p* > .99. The 48 monochrome picture pairs matched the words (e.g. ‘hospitalised’—a picture of a person in a hospital bed; ‘sweeping’—a picture of a person sweeping the floor). The neutral halves of the picture-pairs matched the threat pictures in content and complexity (e.g. doctors performing surgery—people playing a board game).

Six people with asthma and 7 healthy individuals who did not take part in the visual probe task, rated the relevance of the health-threat stimuli to illness in general on a scale from 0—not at all relevant to 10—extremely relevant. The mean ratings were 6.9 (*SD* = 1.4) for words and 6.1 (*SD* = 1.7) for pictures. The ratings for activity/exercise were 6.1 (*SD* = 0.6) for words and 6.3 (*SD* = 0.5) for pictures.

### Data reduction and analysis

The Reaction Time (RT) (in milliseconds) data were filtered by removing RTs for incorrect answers, for responses below 200 ms and above 2000 ms, and those more than 2 *SDs* from that participant’s mean (Lees et al., 2005). Groups did not differ on accuracy rates and amount of data included in the analysis, all *p* > .30.

Attention bias scores were calculated following the formula ((TUDL – TLDL) + (TLDU − TUDU))/2, where T—threat, N—neutral stimulus, U-upper and L-lower denoted the position above or below the fixation point, and D was the target arrow (Asmundson & Hadjistavropoulos, 2007). The positive values indicated an attentional bias towards threat stimuli, and negative values indicated attentional avoidance.

Congruence and Incongruence scores for the visual probe task were calculated using the formula suggested by (Asmundson & Hadjistavropoulos, 2007). The procedure involved subtracting RTs on congruent threat trials from those recorded on the neutral trials. Positive values on the Congruence index reflect faster RTs towards threat on congruent trials, indicating attentional capture by the threat word/picture ((NUDU + NUDL + NLDU + NLDL)/4) − ((TUDU + TLDL)/2).

Incongruence scores were calculated by subtracting RTs on incongruent neutral trials from incongruent threat trials, following the formula ((TUDL + TLDU/2) − ((NUDU + NUDL + NLDU + NLDL)/4)). Positive scores suggest difficulties disengaging from threat, reflecting slower RTs on incongruent threat-neutral trials than on neutral–neutral trials, indicating a difficulty in switching attention from a threat towards the arrow appearing in place of a neutral stimulus.

The effect of mood manipulation was checked by conducting two 2 × 2 ANOVAs with induction (depressed, neutral) as the between subjects and time (pre-induction, post-induction) as the within-subjects variable. Positive and negative affect scores (PANAS) were the dependent variables. Significant interactions were clarified by conducting paired-samples *t*-tests. To check if the induction affected each group differently a three-way 2 × 2 × 2 ANOVA factorial design was conducted with group and induction as between-subjects and time as within-subjects variable.

The attentional bias scores from the visual probe task were entered into a four-way 2 × 2 × 2 × 3 factorial design ANOVA with group (asthma, control) and induction (depressed, neutral) as between-subjects independent variables. Stimulus (words, pictures) and threat type (health-threat, activity, neutral) were repeated measures. Significant interactions were clarified using independent samples *t* tests. Correlational analysis was conducted to test the association between self-report measures and attentional bias scores. Independent correlation coefficients were compared using z-scores and two-tailed tests of significance (Lee & Preacher, 2013).

### Equipment

The computer tasks were run on a desktop computer (CTX Model: PR711T, CTX Ultra Screen Monitor, 17-inch screen) and a Toshiba Satellite A100 laptop (15.4-inch screen). The presentation of the task and data collection was conducted using E-Prime software version 1.1 (SP3) (1.1.4.4). Participants sat in a comfortable position in front of the computer screen. The distance between the participant and the screen was approximately 50 cm. Previous research suggested that any variation in distance between the participant and computer screen had no significant influence on the effect (Owens et al., 2004).

### Procedure

Within each group (45 asthma, 39 control) participants were randomly allocated to undergo either depressed or neutral mood state induction. A random numbers list was used to allocate participants, where odd numbers were assigned to depressed mood and even numbers to neutral mood. Thus, there were 22 asthma participants and 21 controls allocated to the depressed mood induction, and 23 asthma and 18 control participants allocated to the neutral mood induction.

Participants were tested alone in a single session lasting approximately 1 h. The investigator explained the procedure before obtaining written consent. Participants completed the mood induction, then the visual probe task, and self-report measures at the end. Participants were debriefed and compensated for their time (£10).

### Power analysis

Sample size for the current study was calculated using G*Power, version 3.1.3 (Faul et al., 2007, 2009). For the parameters of the power set at .95 and alpha set at .05 a total sample size of 80 participants would be needed to detect a medium effect size, Cohen’s *f* = .25, for the ANOVA of bias scores 4-way interaction with between measures of group (control, asthma) x induction (depressed, neutral), and within measures of stimulus (words, pictures) and threat type (health-threat, neutral) (Cohen, 1992).

## Results

### Mood manipulation check

The analysis of negative affect scores demonstrated a significant induction*time interaction *F*(1,82) = 13.05, *p* = .001, η_p_^2^ = .137. Negative affect increased significantly following the depressed mood induction *t*(42) = 3.19, *p* = .003 the mean difference was 2.5 (*SD* = 5.2).

Positive affect scores also showed a significant induction*time interaction *F* (1,82) = 7.10, *p *= .009, η_p_^2^ = .080. Positive affect decreased significantly after the depressed mood induction; the mean difference was 6.1 (*SD* = 5.9), *t* (42) = 6.77, *p* < .001. Positive affect also decreased significantly after the neutral mood induction, but to a lesser degree; the mean difference was 3.0 (*SD* = 4.6), *t* (40) = 4.16, *p* < .001.

The ANOVA with the added group (asthma, control) factor did not show a significant three-way interaction group*induction*time, with *p* > .98 for negative, and *p* > .78 for positive affect, indicating the induction had similar effect on both groups.

### Attentional bias

The ANOVA revealed a significant four-way interaction of group*induction*stimulus*threat *F* (2, 160) = 4.69, *p* = 0.010, η_p_^2^ = .055, 90% CI [0.008, 0.11] and a significant group*induction interaction *F* (1,80) = 9.46, *p* = .003, η_p_^2^ = .106, 90% CI [0.02, 0.22] but there were no significant main effects or lower level interactions, all *p* > .250.

To clarify the four-way interaction two 3-way ANOVAs were conducted separately for linguistic and pictorial stimuli. No significant main effects or interactions were detected for linguistic stimuli, all *p* > .250. In contrast, for the pictorial stimuli there was a significant three-way interaction of group*induction*threat *F* (2, 160) = 4.69, *p* = .010, η_p_^2^ = .055, 90% CI [0.008, 0.11] and similarly, there was a significant two-way group*induction interaction *F* (1,80) = 8.32, *p* = .005, η_p_^2^ = .094, 90% CI [0.02, 0.20].

**Fig. 2:**
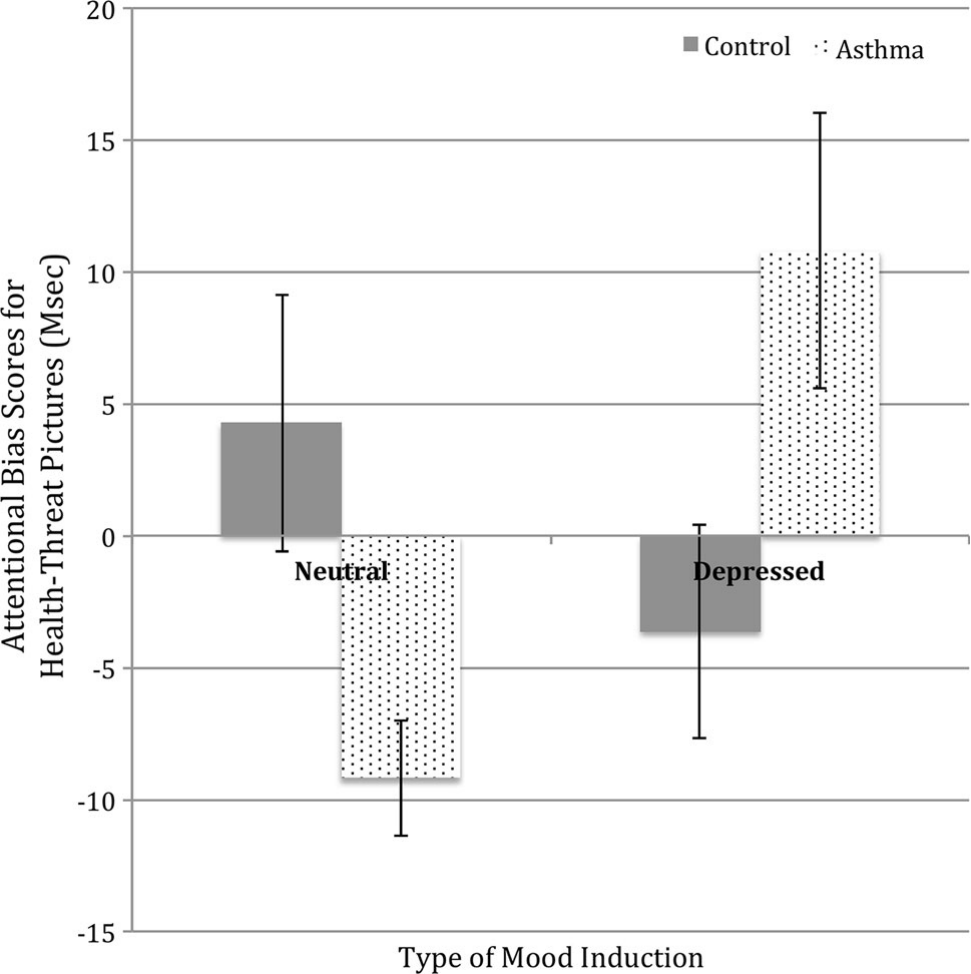
Attentional bias scores for health-threat pictures for asthma and healthy control groups in neutral and depressed mood states

Further analyses to deconstruct the above 3-way interaction were conducted separately for the health-threat and activity pictures. A univariate ANOVA for the activity pictures showed no significant main effects or interactions, all *p* > .250. In contrast, ANOVA for health-threat pictures showed a significant group*induction interaction, *F* (1, 80) = 11.08, *p* = .001, η_p_^2^ = .122, 90% CI [0.03, 0.24]. Most importantly, independent samples *t*-tests revealed that in a neutral mood, the asthma group showed significant attentional avoidance of health-threat pictures (*M* = − 9.20, *SD* = 10.43) compared to the control group (*M* = 4.26, *SD* = 20.64), *t* (39) = 2.72, *p* = .010, *d* = .883, 95% CI [− 5.57, 3.81]. However, the asthma group in a depressed mood showed a significant attentional bias towards health-threat pictures (*M* = 10.77, *SD* = 24.53) compared to the control group (*M* = − 3.63, *SD* = 18.60), *t*_41_ = 2.16, *p* = .037, *d* = .676, 95% CI [− 5.69, 7.05] (see Fig. 2, Table 2).

**Table 2:** Attentional indices for health-threat for asthma and healthy controls

	Depressive mood		Neutral mood	
	Asthma	Control	Asthma	Control
	*M* (SD)	*M* (SD)	*M* (SD)	*M* (SD)
Health-Threat Word Bias	2.8 (16.3)	0.9 (19.8)	− 0.0 (16.4)	0.4 (22.7)
Health-Threat Picture Bias	10.8 (24.5)	− 3.6 (18.6)	− 9.2 (10.4)	4.3 (20.6)
Health-Threat Word Congruence	− 0.2 (13.8)	0.8 (18.7)	− 3.1 (15.7)	1.9 (18.6)
Health-Threat Picture Congruence	− 2.0 (18.9)	− 7.4 (16.3)	− 12.9 (19.6)	− 2.9 (16.7)
Health-Threat Word Incongruence	3.0 (19.2)	0.1 (17.4)	3.1 (14.9)	− 1.6 (18.3)
Health-Threat Picture Incongruence	12.8 (17.8)	3.8 (15.3)	3.7 (15.8)	7.2 (20.2)

Positive bias scores reflect attentional approach

Negative bias scores reflect attentional avoidance

Positive engagement scores reflect attentional capture by the threat stimuli

Positive disengagement scores reflect difficulty in disengaging attention from threat

Analysis within each group revealed that people with asthma allocated to the neutral mood induction showed attentional avoidance compared to those allocated to the depressed mood induction who demonstrated attentional bias towards health-threat pictures, *t* (43) = 3.58, *p* = .001, *d* = 1.093 95% CI [− 6.43, 4.25]. That was not the case for the control group, *p* > .250.

### Congruence and incongruence

In order to explore the magnitude of potential attentional engagement or disengagement difficulty within the asthma group in depressed mood one sample *t*-tests were conducted to compare each mean against zero, where zero signifies an absence of the bias either towards or away from Health-Threat pictures or words (Gotlib et al., 1988).

For the congruence scores, one sample *t*-tests revealed that neither Health-Threat words or Health-Threat pictures were significantly different from zero, *p* > .60. For the incongruence scores, the contrast was not significant for the words, *p* > .40, but the difference from 0 was significant for the Health-Threat pictures, *t* (21) = 3.37, *p* = .003, 95% CI [4.9, 20.7], *d* = 0.72, 95% CI [0.23, 1.19]. This suggests that potentially the attentional bias is underpinned by the difficulty in disengaging attention from Health-Threat pictures experienced by the asthma group induced into depressed mood.

The paired-samples comparison between congruence and incongruence scores for the Health-Threat pictures within the asthma depressed group similarly showed a significant difference, *t*(21) = 2.54, *p* = .019, 95% CI [− 26.9, − 2.7], Hedges *g* = 0.78, 95% CI [0.14; 1.45].

### Correlations between attention bias scores and self-report measures

Correlational analysis of attentional bias scores and asthma duration and severity showed that there was a significant positive association between asthma severity level and attentional incongruence scores for Health-Threat words *r*_45_ = .32, *p* = .033 and for Health-Threat pictures *r*_45_ = .31, *p* = .041. These results indicate that greater asthma severity is related to greater difficulty in disengaging attention from health threatening stimuli. The correlations between illness duration or asthma interference scores and attentional indices were not significant, all *p* > .10.

Correlational analysis of anxiety scores (HADS) and attentional bias scores was conducted to determine whether biased attention in asthma might have been due to elevated anxiety. In the neutral mood, two correlations showed a trend towards significance, but more importantly showed diametrically opposing patterns. For the asthma group in neutral mood, greater attentional bias towards health-threat pictures was associated with lower anxiety, *r*_22_ = − .42, *p* = .052. In contrast for the control group in neutral mood greater attentional bias was associated with higher anxiety *r*_18_ = .43, *p* = .074. Comparison of these two correlations showed a significant difference *z* = 2.63, *p* = .008. The equivalent correlations were not significant in the induced depressed mood group.

## Discussion

The current study investigated attentional processing in people with asthma in an induced depressed or neutral mood. In support of the first hypothesis, the asthma group in an induced depressed mood showed an attentional bias towards health-threat pictures, compared to the asthma group in a neutral mood and to the healthy controls in either a depressed or a neutral mood. The results also indicated that this attentional bias may be due to difficulty disengaging from health threat, as opposed to hypervigilance towards health threat.

The significant association between depressed mood and attentional bias in asthma is in line with previous findings (De Peuter et al., 2007; Jessop et al., 2004; Lowther et al., 2015; Martinez-Moragon et al., 2003; Put et al., 2004). The findings in our study, that depressed mood leads to attentional bias towards health-threat and the significant correlation between attentional bias, specifically attentional disengagement difficulties, and self-reported asthma severity, provide support for the proposal that depression may affect the accuracy of symptom perception via attentional mechanisms, and further that symptom over-perception may be the result of biased attention towards internal and external cues beyond asthma-specific information.

Even though these findings are generally in line with previous literature, there are some important differences between the current results and the results of previous studies that need to be addressed. De Peuter et al (2007) did not detect an attentional bias in asthma using a visual probe task, even though they did find a significant association between negative affect and attentional bias.

The key difference in this case may be the stimuli used in the two studies. De Peuter and colleagues’ task included asthma-specific words while the current study demonstrated a bias towards general health-threat pictures. It might have been the combination of depressed mood and health-threat images representing greater threat severity and consequently activating the relevant illness schema, that resulted in increased attentional bias. The linguistic stimuli used by De Peuter and colleagues might have lacked ecological validity and salience for their participants and therefore failed to elicit attentional bias.

However, it is important to note that Lowther et al. (2015) did find an attentional bias towards asthma-related words. Yet, there are key differences between De Peuter et al. (2007) and Lowther et al. (2015) studies. First, the former tested adults and the latter tested children. Age may be important not only with regard to general development, physical and cognitive, but also with regard to the stage of the illness. It is more likely that children have been diagnosed with asthma relatively recently and are still in the process of developing cognitive strategies related to perceiving and coping with these symptoms. Adults with long-term asthma, on the other hand, may already have a long-established illness schema and ways of cognitively processing symptom-related information. The second important difference between the two previous studies was that De Peuter and colleagues used 500 ms stimulus duration while Lowther et al used 1250 ms deemed more appropriate for children. This introduces the possibility that De Peuter et al were tapping into earlier automatic attentional stages, whereas Lowther et al were tapping into the later elaborative stages.

Regarding the difference between Lowther et al’s finding of a bias towards words and the current study’s failing to find one, this may also be due to stimulus duration. Linguistic stimuli may need longer processing time than pictures, thus the current study might have detected an attentional bias towards the words if they were presented for longer than the 500 ms employed. Furthermore, Dudeney et al. (2017) failed to detect an attentional bias towards asthma-related words in children, in contrast to Lowther et al. However, Dudeney et al propose that might have been due to differing asthma severity levels; Lowther et al were dealing with a sample of children with more severe asthma. The significant association between attentional bias and asthma severity detected in the current study provides additional support for the above proposal.

Some tentative conclusions can be reached based on the above discussion of the findings from the four studies that have used the visual probe task, including the current one. The magnitude of attentional bias in asthma may be impacted by the following factors: depressed mood is likely to strengthen the bias; threat severity, where pictures of health-threat contribute to the increase in attentional bias; symptom severity, where greater asthma severity is likely to be accompanied by stronger bias; information type, where pictures may activate the illness schema at an earlier stage of processing than linguistic stimuli.

As far as we are aware there are no other studies that manipulate mood in asthma and examine the possibility of consequent attentional bias. However, the potential influence of naturally occurring differences in level of depressed mood has been examined by Jessop et al. (2004) using a Stroop task. They failed to find a significant relation between Beck Depression Inventory scores and speed of colour categorisation of asthma-related words, perhaps because of a reliance upon response interference in the Stroop task. Consistent with this, extensive research has demonstrated a lack of correlation between attentional biases in the Stroop and visual probe tasks (Asmundson et al., 2005; Dalgleish et al., 2003; Gotlib et al., 1988), suggesting that the tasks are tapping into non-overlapping processes (e.g., response inhibition in the Stroop task and attentional allocation in the dot-probe task).

The current results demonstrate for the first time that attentional bias in asthma is directly influenced by depressed mood and may generalise beyond asthma-specific stimuli to general health-threat stimuli. The findings provide support for a theoretical model that proposes that depressed mood leads to attentional bias towards symptoms (even those unrelated to asthma itself) and thus potentially the conferral of exaggerated importance upon minor symptoms and a consequent diminution in levels of fine control over asthma (Janssens et al., 2009). Therefore, attentional bias may undermine the accuracy of symptom monitoring, resulting in symptom over-perception.

The consequences of such over-perception, however, are less clear. It may, for example, result in increased catastrophizing, fear, and panic, which in turn would trigger asthma symptoms and lead to more hospital visits and greater health-care utilization. However, it would be more likely that catastrophic cognitions and fear would be associated with hypervigilance. The current results point more towards difficulty in disengagement which would be a more natural consequence of depressed mood which tends towards rumination, elaboration, and dwelling rather than hypervigilance.

In order to predict the potential consequences of depressed mood-induced attentional bias towards health-threat, it is important to carefully consider the nature of such negative mood and the content of health-threat information used in the current study. The depressed mood induction in this study increased negative affect as measured by the Positive and Negative Affect Scale (Watson et al., 1988). The negative affect subscale covers a variety of emotions, including fear, anger, and sadness, which extend beyond strictly depressed mood.

The health-threat stimuli were also quite varied, including words relevant to disease (e.g. cancer), treatment (e.g. hospitalisation), disability (e.g. paralysed), illness consequences (e.g. dying), covering a range of potential health-threat. Any of these concepts could be key in biasing attention in negative mood, and different concepts may lead to different consequent cognitive, emotional and behavioural processes. Anxiety and fear may lead to an increase in vigilance and catastrophizing in relation to minor physical sensations. Anger and irritability may lead to increased rumination and brooding, hostility, and undermine treatment compliance. Sadness and hopelessness may lead to increased rumination, difficulties in shifting attention away from symptoms or health-threat, and undermine beliefs in treatment effectiveness. Distinguishing maladaptive attentional bias towards more general health-threat from beneficial amplified attention towards asthma-specific information may be aided by further investigating the exact nature of the information that consumes attentional resources and the nature of its interaction with specific types of emotional states.

In contrast to the hypothesis that attentional bias, albeit of a smaller magnitude, would be observed in the neutral mood, participants with asthma demonstrated attentional avoidance of health-threat pictures following neutral mood induction. The result, though not predicted, echoes similar findings from other studies in respiratory disorders. Dudeney et al. (2017) detected avoidance of asthma-related stimuli among children with asthma and in children with comorbid asthma and anxiety, as indicated by the negative values of the attentional bias index. Livermore et al. (2007) also found that people with chronic obstructive pulmonary disorder comorbid with panic disorder attended away from threat words.

One explanation of the avoidance of health-threat demonstrated in the neutral mood asthma group may have to do with the nature of the mood induction. The neutral mood induction consisted of Velten statements, for example, participants were instructed to think about a statement “Boeing’s main plant in Seattle employs 35,000 people”. The induction appeared to be successful, because the positive mood was reduced afterwards, as would be expected when we are trying to induce a neutral mood state. However, Velten neutral mood induction is similar to a distraction induction developed for the purposes of altering a cognitive state (Nolen-Hoeksema et al., 1993; Watkins & Teasdale, 2001). The distraction induction includes similar types of statements that describe neutral external matters and concrete images of buildings, transport, or nature. The neutral mood induction in the current study might have succeeded not only in altering mood but also cognition, putting participants in a distracted cognitive state and that might have resulted in active avoidance of health-threatening stimuli.

The current results are also consistent with previous findings of increased use of repressive cognitive styles, denial, and avoidant coping in asthma (Adams et al., 2000; Cooke et al., 2003; Gonzalez-Freire et al., 2010). Repressive coping, defined as avoidance of conscious perception and recall of negative feelings (Weinberger, 1995) is one of the barriers to better symptom control and management. Repressive coping is strongly linked to poorer lung function and greater symptom severity, negative health outcomes and increased utilization of health care in asthma (Adams et al., 2000; Cooke et al., 2003; Gonzalez-Freire et al., 2010). The attentional avoidance shown by the asthma group in neutral mood may reflect these types of maladaptive monitoring strategies which might have been triggered by this kind of neutral/distraction induction. Avoidance and under-monitoring may result in symptom under-perception also compromising asthma management (Janssens et al., 2009).

For that matter, the finding of the significant negative correlation between attentional bias and anxiety in the asthma group in neutral mood can be interpreted in different ways. Increased attention towards health threat associated with a decrease in anxiety may indicate that a certain degree of monitoring can be adaptive in the context of chronic illness or in the presence of an actual threat. It may be needed to aid in accurate symptom perception, and may decrease health-related anxiety. This result seems to be in line with recent findings that a certain degree of cognitive bias towards threat can improve health-related behaviour via the mechanism of increased worry (Notebaert et al., 2014). While that may seem contrary to our result showing decreased anxiety accompanying attentional bias, current research and theoretical directions suggest that there may be different mechanisms involved in adaptive worry as opposed maladaptive worry or anxiety (Macleod, 2012; Notebaert et al., 2014). Therefore, future research should explore further under what conditions attentional bias may be adaptive or maladaptive within the context of a chronic illness and the interaction between health-related or general worry, anxiety and cognitive processing.

Alternatively, the negative correlation between attentional bias and anxiety may also be interpreted as evidence that increased anxiety contributes to avoidance. The harmful role of avoidance is well-documented in the anxiety literature (Cisler & Koster, 2010). Therefore, the potential link between anxiety and avoidance in asthma is unlikely to be an aspect of adaptive monitoring in the case of asthma. In contrast, such an association may undermine adaptive monitoring and effective symptom management.

As is the case with psychological disorders, such as depression, difficulty in attentional disengagement and avoidance of health-threat modulated by mood may have similar clinical relevance for the management and control of asthma. A cognitive task, like the visual probe task in the current experiment, could be adapted for clinical purposes with regard to asthma management. Firstly, this easily administered task could aid patients explore and become more aware of their own symptom monitoring patterns and the influence of their current mood. Secondly, attention can be trained to focus on relevant asthma-related information or away from the negative distressing general health-threat (Hertel & Mathews, 2011; Macleod, 2012; Notebaert et al., 2014). Using pictures, particularly those that are salient and ecologically valid stimuli, would increase the efficacy of the attentional task.

A recent study demonstrated a potential effect of changing the attentional focus on reducing excessive coughing in healthy volunteers (Janssens et al., 2014). The current proposed attentional training paradigm may be similarly effective in addressing inaccurate perception of asthma symptoms (Janssens et al., 2009). Modulating attention for the purposes of more accurate symptom assessment may also reduce the rate of non-adherence to medication in asthma that arises from under-perception of symptoms (Janssens et al., 2012).

Attentional bias modification techniques, however, are still in their infancy, and their actual effectiveness at the moment is under discussion (Bar-Haim, 2010). There is also an issue of the reliability of the visual probe paradigm when it comes to detecting individual level variations in attentional processing (Schmukle, 2005). Moreover, while the current evidence does not refute the presence of attentional bias in asthma, its specific nature is far from being fully explored, therefore interventions that involve attention modification should be approached with caution.

There are other techniques that could benefit from further investigation of the nature of attentional bias in asthma. For example, interventions that aim to enrich and improve attentional processing and monitoring thereby helping to regulate cognition and mood, rather than modifying the content of negative cognitions or negative schema. Considering that the question of whether the attentional bias in asthma is adaptive or counterproductive is unresolved, interventions that help gain control over attentional monitoring as opposed to modifying its course may be more appropriate. Metacognitive therapy in social phobia is an example of one such technique. It attempts to modify an overly self-focused attentional strategy that may lead to hyperawareness of symptoms, consuming cognitive resources, thereby diverting attention away from other information that may disconfirm catastrophic cognitions (Wells, 2007). The aim of such treatment is not simply to change the attentional focus, but to expand it beyond the self, redirecting it towards external information. The technique aims to improve attentional flexibility which would allow attentional shifts between internal and external, or social and non-social information. This may prove to be particularly useful in the case of asthma, where simply reducing attention towards symptoms may be harmful as it would lead to under-perception of symptoms. Improving attentional control and range, however, may contribute to adaptive monitoring, increase perceptual accuracy, and aid in mood regulation, which would in turn prevent the attentional bias towards symptoms from getting out of control and turning into hyperawareness and catastrophizing.

Mindfulness Based Cognitive Therapy also aims to help people to learn to become aware of their thoughts, feelings and bodily sensations in a non-judgemental way. The advantage of mindfulness may be that it may help repair and control attentional processes rather than modify their course or change their content. Mindfulness techniques do the opposite of reducing attentional bias, but rather help increase awareness and improve perception of feelings, thoughts, physical sensations, as well as increasing the accuracy of discrimination between different cognitive, affective, and physical experiences (Williams et al., 2008). A recent mindfulness trial found that mindfulness techniques can improve the quality of life and decrease stress in asthma patients (Pbert et al., 2012). Even though the intervention did not improve objective lung functioning, it showed the potential for mindfulness to introduce lasting improvement in perceptual awareness that can aid in accurate symptom perception and symptom control (Pbert et al., 2012).

The current study has a number of limitations, specifically regarding the sample, asthma measurements, and certain aspects of the cognitive task. The sample of asthma patients was drawn from a wider community rather than any specific asthma clinic; thus the self-reported asthma diagnosis was not confirmed by a physician, and for the majority of the sample, asthma had been diagnosed in childhood. The study also lacks objective measurements of asthma symptoms and lung function, relying instead on self-reports. The current asthma group may also be quite well adjusted and high-functioning as reflected by their relatively mild reported asthma severity, and low emotional distress as indicated by their low levels of anxiety and depression. The group’s high educational level may also be a contributing factor to their high levels of functioning. Furthermore, their educational level may be a factor in the groups’ perception and processing of the linguistic stimuli in the visual probe task. Thus, we cannot generalise the current results to various subpopulations within the wider population of people with asthma, such as those who were diagnosed recently, or those who suffer from more severe asthma.

There is also an important potential limitation with regard to the type of stimuli used in the visual probe task. The study did not include asthma-related pictures and words thus limiting comparison of the current results with the previous literature on attentional bias in asthma which used asthma-specific words rather than general health-threat words and pictures. Even though the aim of the current study was to investigate attentional processing of more general threat rather than illness-specific, it would be important to include respiratory stimuli as another condition. This is one of the problems of tasks that measure reaction time, in order to improve measurement reliability, the task has to include a large number trials. The visual probe task in the current study was rather long, taking on average 14 min, which was tiring for some participants. In particular, people may lose focus and motivation, which may be detrimental to their performance reducing the amount of data available for analysis if participants start making errors. Future research could take this into account, by breaking the task up into shorter blocks. That would allow inclusion of a good number of relevant conditions, for example different types of stimuli (illness-specific, general health-threat, negative emotion, physical or social threat, depression or anxiety specific stimuli) or varying the exposure durations in order to explore the time course of attention (100, 500, 750 ms or above 1000 ms stimulus duration).

The mood manipulation in the study was successful. Depressed mood induction led to increased negative affect and reduced positive affect. However, negative affect (measured by items such as “upset”, “irritable”, “scared” which indicate negative aspects of mood states in general) is not the same as depressed mood, and depressed mood is not depression. Therefore, it is too early to make conclusions about the effect of clinical levels of depression or of naturally occurring depressed mood on attentional bias in asthma. The study also did not include other moods for comparison, such as, a positive mood or anxious mood, again making it difficult to make firm conclusions about the role of depressed mood specifically rather than negative affect in general. It may be that it is the general elevation in emotional level, greater emotional arousal, that effected the attentional bias, compared to a dampening of emotional level resulting from the neutral mood induction. A future study could compare a group of asthma patients with comorbid depression with a group of asthma patients without comorbid depression, or measure naturally occurring depressed mood among people with asthma.

The current findings indicate a presence of potentially maladaptive attentional patterns in asthma, characterised by attentional bias towards health-threat in depressed mood and by avoidance of health-threat in neutral mood. This distorted attentional pattern may be the mechanism behind inaccurate symptom perception in asthma. Attentional training could be utilized to improve the accuracy of symptom perception. This in turn may have a wider impact, for example in decreasing stress and anxiety, improving adherence to prescribed medication, reducing the burden on health services, and increasing patients’ well-being and quality of life.
